# St. Mary's Hospital

**Published:** 1905-05-06

**Authors:** Herbert J. Allcroft, Alfred de Rothschild, G. A. Webbe, H. A. Harben, James R. Mellor, W. H. Broadbent, D. B. Lees, Herbert. W. Page

**Affiliations:** Treasurer; Treasurer; Treasurer; Chairman; Deputy Chairman; Consulting Physician; Senior Physician; Senior Surgeon


### ST. MARY'S HOSPITAL.

£60,000 REQUIRED.

The following official statement has been issued in anticipation of the meeting to be held at the hospital on the afternoon of May 9th at 4.30 p.m.:—

For more than half a century St. Mary's has provided hospital relief for the poor of Paddington, West Marylebone, North Kensington, Shepherd's Bush, Acton, Kensal Green, Kilburn, Willesden, and Harlesden.

The population of this district has increased in 53 years from 162,000 to 527,000, but there has been no corresponding increase in the size of the hospital, which now contains only 281 beds, being 80 less than the district was estimated to require when the hospital was built in 1851. Notwithstanding the addition made to the hospital in 1884, the need for increased accommodation has been constantly felt, and it has been found impossible to provide for the ever-increasing numbers of poor people requiring treatment, many of whom have had, in consequence, to be turned away. In addition to the pressing necessity for enlargement came the need for improvement in the internal arrangements of the hospital. The great advance which had taken place in the science of hygiene showed that arrangements regarded as satisfactory in 1851 were, as a matter of fact, seriously defective. The appointments and equipment of the hospital had become obsolete, and the inadequacy of the accommodation provided in the kitchen, laundry, nurses' home, and servants' quarters made it impossible to carry on efficiently the work of the service departments of the hospital. The Governors therefore decided, so long ago as 1888, to undertake the work of enlargement and improvement.

Four years later, on December 17th, 1892 (an adjoining site having meanwhile been purchased and cleared at a cost of nearly £40,000), his Majesty the King, then Prince of Wales, laid the foundation-stone of the Clarence Memorial Wing. The means at the command of the hospital, however, only allowed of the completion of the basement, containing the new out-patients' department. This was opened in 1898, after which time the building work was suspended for four years for lack of funds. Encouraged by a bequest of £25,000 from the late Mr. Zunz, the board of management resumed the work in 1902, and the main structure of the Clarence Memorial Wing is now nearly finished, so nearly, indeed, that there is no alternative but to complete and warm it in order to prevent deterioration, while it has been found necessary, in the interests of the nursing staff, to occupy the three upper floors as a nurses' home.

At this crucial point, when the works cannot be suspended, not only has the special fund raised for building purposes become exhausted, but, during 1904, owing to a falling off in the amount of legacies, there was a deficiency of no less than £10,000 in the general maintenance fund.

This is the serious financial situation which now confronts the hospital.

The completion of the Clarence Memorial Wing, now in progress, will cost £20,000.

The remodelling and refitting of the service departments in the basement of the old building, upon which the heating, hot water supply, power for lifts, etc., in the new wing depend, will cost £15,000 more.

Before the wards and theatres in the new wing can be used they must be furnished, and extensive alterations in the old hospital are indispensable in order to combine the old and the new into a complete modern hospital on the scale of 340 beds. This will, it is estimated, cost a further £15,000.

It will thus be seen that to discharge existing encumbrances and to complete the work of enlargement and improvement a sum of £60,000 is immediately required, while an increased income will be necessary to maintain the additional 60 beds which the new wing will accommodate.

Unless this £60,000 is forthcoming there will be no alternative but to sell the investments of the hospital, representing the accumulated savings of half a century, and amounting only to £46,000.

If this becomes necessary, the resulting loss of income will not only prevent the opening of the Clarence Memorial Wing, but will also compel the closing of some of the wards of the old hospital.

Thus the chief object of these 12 years of strenuous effort will be frustrated, the large sums which have been spent on the building of the new wards will be lying unproductive, the 60 beds, which would in the course of the year provide for the treatment of 1,400 sick poor, will be left empty, and the most important branch of the work of the hospital—the relief of in-patients—instead of being largely extended, will be actually curtailed.

The board of management feel confident that the public will not permit such a calamity to happen if the facts are brought to their notice, and they therefore make an earnest appeal for help.

Contributions may be sent to any of the treasurers, or to the secretary, at the hospital, or to the London and County Bank, Paddington branch.

Herbert J. Allcroft,
Alfred de Rothschild, } Treasurers.
G. A. Webbe,
H. A. Harben, Chairman.
James R. Mellor, Deputy Chairman.
W. H. Broadbent, Consulting Physician.
D. B. Lees, Senior Physician.
Herbert W. Page, Senior Surgeon.

